# Tuberous sclerosis, deep vein thrombosis and lack of C and S proteins: A case report

**DOI:** 10.12861/jrip.2012.12

**Published:** 2012-01-01

**Authors:** Seyed Seifollah Beladi-Mousavi, Abbasali Zeraati, Farzaneh Sharifipour, Masih Naghibi, Farnaz Kalani, Sara Moussavinik, Marzieh Beladi-Mousavi

**Affiliations:** ^1^Department of Internal Medicine, Faculty of Medicine, Jundishapour University of Medical Sciences, Ahvaz, Iran; ^2^Department of Nephrology, Imam Reza Hospital, Mashhad University of Medical Sciences, Mashhad, Iran; ^3^Department of Chemistry, Behbahan Branch, Islamic Azad University, Behbahan, Iran

**Keywords:** Tuberous sclerosis, Adult polycystic kidney disease, Protein C, Protein S

## Abstract

**Introduction:** Tuberous sclerosis, an autosomal dominant disorder, is characterized by hamartomas in different organs of body. Kidney involvement is quite common in this disorder and sometimes it is accompanied by adult polycystic kidney disease.

**Case:** A 46-year-old woman who was being treated for adult polycystic kidney disease and systemic hypertension was admitted to this hospital because of acute lower limb edema. Color Doppler sonography study showed deep vein thrombosis of lower limbs and also left iliac vein. Despite the initiation of hourly heparin infusion, the patient involved by pulmonary emboli on the 2^nd^day of admission. Lab tests revealed protein C and S deficiency. The patient had already experienced episodes of pneumothorax too. Cutaneous lesions due to sebaceous adenoma were seen on her cheeks, nose and neck. She had also periungual fibroma suggestive of tuberous sclerosis.

**Conclusion:** Although, according to our patient with both tuberous sclerosis and protein C and S deficiency, a significant relation between these two diseases, cannot confirmed, however, evaluation of other patients who have tuberous sclerosis can help to confirm or rule out this relation.

**Implication for health policy/practice/research/medical:**

Tuberous sclerosis, an autosomal dominant disorder, is characterized by hamartomas in different organs of body. Kidney involvement is quite common in this disorder and sometimes it is accompanied by adult polycystic kidney disease. Regarding the few number of patients with both tuberous sclerosis and protein C and S deficiency, a significant relation between these two, cannot be obviously found. Evaluation of the patients who have tuberous sclerosis can help to confirm or rule out this relation and its etiology.

## Introduction

Tuberous sclerosis is a hereditary autosomal dominant disorder (1,2). It is characterized by hamartomas in various organs of body (1,2). Two genes are known to be responsible for the disease. TSC1, and TSC2 which are located on chromosomes 9 and 16, respectively (3,4). With a low incidence, TSC2 gene can be accompanied by PKD1 gene, which results in manifestation of both phenotypes. In these patients, chronic kidney disease is quite common (1-6).

## Case

A 46- year-old woman was admitted to this hospital because of acute edema in lower limbs, which was dominant in the left one. She had been treating with antihypertensive drugs due to polycystic kidney disease and hypertension for four years by the time she was admitted to this hospital. She had a history of left and right pneumothorax 12 years and 3 months earlier, respectively. She also mentioned a history of thrombosis in lower limb 4 years earlier and an ulcer in her right shin following warfarin consumption.

**Figure 1:**
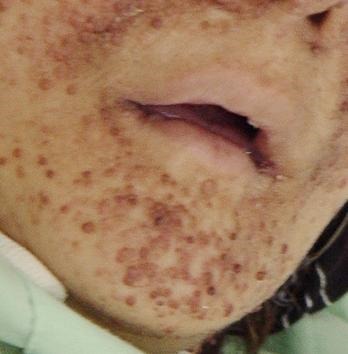
Brown to black papules on her face

**Figure 2:**
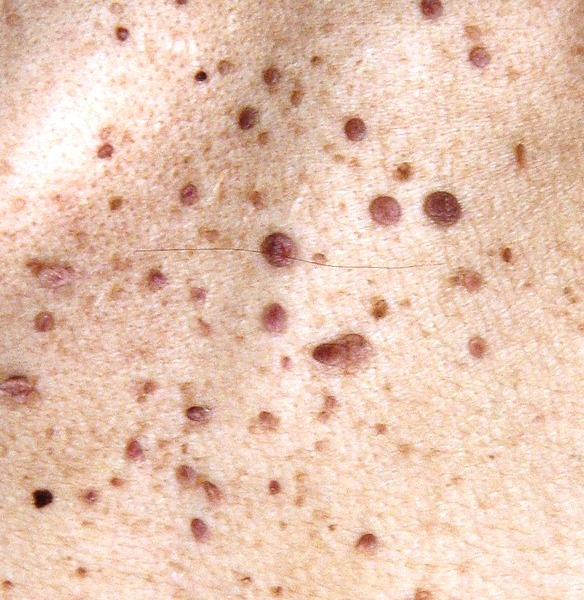
Brown to black papules on her face

**Figure 3:**
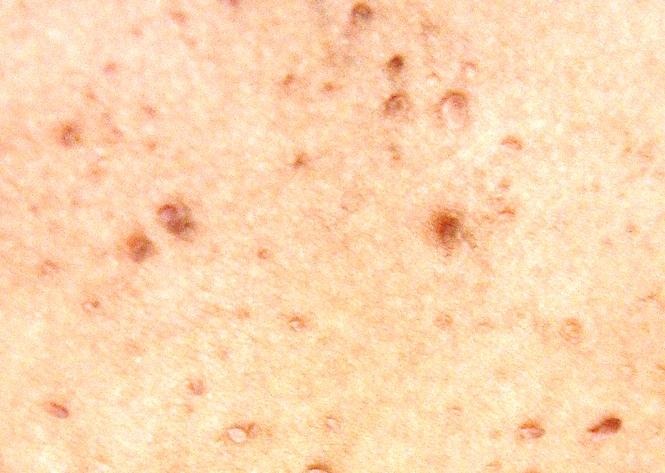
Brown to black papules on her face

**Figure 4:**
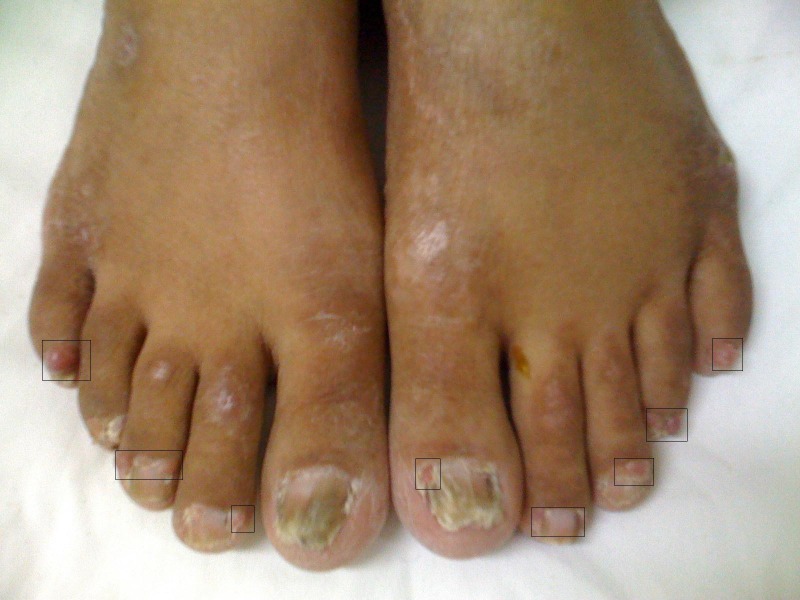
Nail hyperkeratosis and several soft pink nodules and papules around the nails of her hands and feet

There was no history of disease in her parents, two sisters, brother, and her 23 and 26 years old children. Physical examination revealed brown to black papules on her face which were dominant on her cheeks and nose (Figures 1, 2, 3 ). Nail hyperkeratosis and several soft pink nodules and papules were also found around the nails of her hands and feet (Figure 4). Her liver was palpable. She was being treated by metoprolol and amlodipine. Color Doppler sonographic examination of lower limbs and pelvis showed large acute thrombosis in femoral veins of lower limbs and left iliac. Chest radiography revealed fine reticular lesions in the field of both lungs. In lung high resolution CT-scan, minute cystic lesions were noticed. Treatment with hourly heparin infusion was started. On the second day of treatment, the patient got dyspnea. Lung perfusion scan was performed. The diagnosis was pulmonary emboli.

Laboratory tests were as follows:

WBC: 6500 x 103/µL Plt: 164000 /µL, Hct: 37% , PT and PTT were in normal range. AST and ALT: normal Cr: 1.4 mg/dl, Ca: 9.5mg/dl, P: 3.7mg/dl.

D-Dimer: 580ng/ml. Antiphospholipid antibody panel:

negative
Fibrinogen: 413 mg/dl (Normal 200-400)
Protein C activity 48% (72-140)
Protein S activity 29% (60-140)
Anti thrombin III 79% (80-120)
Factor 5 Leiden: negative

An abdominal and pelvic sonographic examination and CT-Scan showed several masses suspicious of hamartomas in liver, and the kidneys were similar to the ones in adult polycystic kidney disease. Pancreas and spleen seemed to be normal. In echocardiography, left ventricular hypertrophy was found and the pulmonary artery pressure was 40 mmHg. Brain CT scan revealed no abnormalities. Heparin dosage was increased and after, warfarin therapy initiation, the patient was discharged after 3 weeks. The final diagnosis was tuberous sclerosis, lower limb venous thrombosis, and pulmonary emboli.

## Discussion

Tuberous sclerosis is a hereditary autosomal dominant disease (1), however, up to 70% of the cases can result from new mutations (1,7).

The disease occurs in 4-5: 100,000 persons. Each of the 2 genes responsible for the disease is able to reveal all its possible manifestations. TSC2 accounts for more severe ones and the probability of its mutation is higher (1,2,4). Neurologic manifestation of tuberous sclerosis, including epilepsy and low intelligence quotient (IQ), along with sebaceous adenoma in skin make the classic triad of the disease which is seen in less than 50% of the patients. Nowadays the diagnostic criteria for tuberous sclerosis consist of a set of major and minor diagnostic features. Two major criteria or one major criterion and two minor criteria will suffice for the diagnosis of tuberous sclerosis.

*Major criteria include:*

1. Angiomyofibroma of the face
2. Connective tissue nevus
3. Hypomelanotic patches (3 or more)
4. Ungual or periungual fibroma
5. Lymphangiomyomatosis
6. Renal angiomyolipoma
7. Cardiac rhabdomyoma
8. Multiple retinal hamartomas
9. Cortical tuber
10. Subependymal giant cell astrocytoma
11. Subependymal nodules

*Minor criteria include:*

1. 1 or 2 hypomelanotic macules
2. Gingival fibromas
3. Multiple pits in dental enamels
4. Multiple renal cysts
5. Hamartomal colon polyps
6. Bone cysts
7. Achromic patch of the retina
8. Cerebral white matter radial migration lines

Kidney involvement is seen in 60-75% of the patients and is more common when TSC2 is present. This involvement includes angiomyolipoma, cyst or renal cell carcinoma. Cysts are seen as 2 types: 1) one or several simple cysts with no symptoms. 2) Tuberous sclerosis along with polycystic kidney disease; in this group cyst are just like autosomal dominant polycystic kidney disease (ADPKD), large and multiple with the symptoms similar to ADPKD. Renal failure is common in these patients (2). On chromosome 16, as the TSC2 gene is adjacent to PKD1 gene, any mutations in TSC2 also affects PKD1, and the phenotype of both diseases will show up (2,4,5). The presented case had pulmonary, hepatic, dermatologic and renal manifestation and the kidney involvement was as the type of PKD in ADPKD. Although genetic evaluation was not performed, regarding the multiple cysts in the kidney, TSC2 considered to be responsible for the disease. The patient was first admitted because of venous thrombosis in lower limbs and later in the following treatment, she got pulmonary emboli. Lab tests revealed protein C and S deficiency, Fibrinogen and antithrombin III were a bit higher and lower than normal, respectively. Protein C deficiency can be hereditary or acquired. Severe infections, septic shock, disseminated intravascular coagulation (DIC), some malignancies, and hepatic disorder (9) can lead to its shortage. But our patient had none of them. Protein C deficiency is autosomal dominant, and the gene is located on chromosome 2 (10); therefore there is no significant genetic relation between the shortage of this protein and tuberous sclerosis. Deficiency in protein S would be in two forms of hereditary and acquired (11). The two homologous genes of this protein, one perfect and active and the other probably a psudogene, are located on chromosome 3 (12). Its hereditary shortage is in the form of autosomal dominant and the disease manifests itself in the form of repeated thrombosis in deep veins and arteries (11). The acquired form occurs in association with HIV infection, liver disease, nephrotic syndrome, DIC, and pregnancy. Protein C deficiency along with tuberous sclerosis has been reported in two cases (13,14). Okafor and colleagues reported a 51-year-old woman with portal vein thrombosis and esophageal varices who had tuberous sclerosis along with protein C deficiency. She had dermatologic and hepatic manifestation. Her spleen was large and she had retroperitoneal angiomyolipoma. No involvement was detected in her kidneys, nervous system and other organs. Later patient experienced venous thrombosis of the right lower limb (13). In Vasudevan and colleagues case report, a 29-year-old woman was presented who had tuberous sclerosis and portal vein thrombosis along with protein C and S deficiency and multiple abortions. The patient had dermatologic, ophthalmic, renal and cerebral manifestations, with no evidence of pulmonary or hepatic involvement (14). The common points among these three patients are as follows: being female, having dermatologic lesions and having angiomyolipoma in various organs (retroperitoneal space, brain and liver).

## Conclusion

Regarding the few number of patients with both tuberous sclerosis and protein C and S deficiency, a significant relation between these two, cannot be obviously found. Evaluation of the patients who have tuberous sclerosis can help to confirm or rule out this relation and its etiology.

## Acknowledgements

The authors wish to graciously acknowledge the efforts of Ms. Toktam Moussavinik in revising the paper.

## Conflict of interests

The author declared no competing interests.

## Ethical considerations

Ethical issues (including plagiarism, informed consent, misconduct, double publication and redundancy) have been completely observed by the authors.

## Funding/Support

None declared.

## Authors’ contributions

SSBM and AZ prepared the primary draft. FS, MN, FK and SM wrote some parts of the manuscript. SSBM and MBM prepared the final manuscript.
